# High-Throughput Mining of Novel Compounds from Known Microbes: A Boost to Natural Product Screening

**DOI:** 10.3390/molecules29133237

**Published:** 2024-07-08

**Authors:** Surya Nandan Meena, Anna Wajs-Bonikowska, Savita Girawale, Md Imran, Preethi Poduwal, Kisan M. Kodam

**Affiliations:** 1 Department of Chemistry, Savitribai Phule Pune University, Pune 411007, India; suryanandanmeena6@gmail.com (S.N.M.); kisan.kodam@unipune.ac.in (K.M.K.); 2 Institute of Natural Products and Cosmetics, Faculty of Biotechnology and Food Sciences, Łódz University of Technology, Stefanowskiego Street 2/22, 90-537 Łódz, Poland; 3 Department of Botany, University of Delhi, Delhi 110007, India; 4 Department of Biotechnology, Dhempe College of Arts and Science, Miramar, Goa 403001, India; biotech.preethi@unigoa.ac.in

**Keywords:** natural product, microbes, biosynthetic gene clusters (BGCs), genome mining, antiSMASH, HiTES, LAESI-IMS, dereplication

## Abstract

Advanced techniques can accelerate the pace of natural product discovery from microbes, which has been lagging behind the drug discovery era. Therefore, the present review article discusses the various interdisciplinary and cutting-edge techniques to present a concrete strategy that enables the high-throughput screening of novel natural compounds (NCs) from known microbes. Recent bioinformatics methods revealed that the microbial genome contains a huge untapped reservoir of silent biosynthetic gene clusters (BGC). This article describes several methods to identify the microbial strains with hidden mines of silent BGCs. Moreover, antiSMASH 5.0 is a free, accurate, and highly reliable bioinformatics tool discussed in detail to identify silent BGCs in the microbial genome. Further, the latest microbial culture technique, HiTES (high-throughput elicitor screening), has been detailed for the expression of silent BGCs using 500–1000 different growth conditions at a time. Following the expression of silent BGCs, the latest mass spectrometry methods are highlighted to identify the NCs. The recently emerged LAESI-IMS (laser ablation electrospray ionization-imaging mass spectrometry) technique, which enables the rapid identification of novel NCs directly from microtiter plates, is presented in detail. Finally, various trending ‘dereplication’ strategies are emphasized to increase the effectiveness of NC screening.

## 1. Introduction

**Figure 1:**
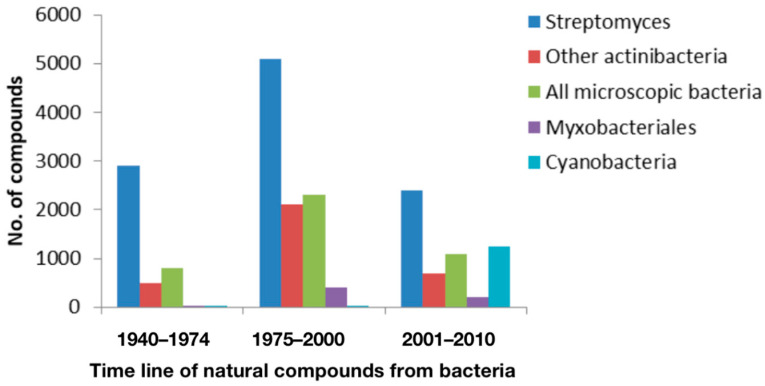
Approximate number of natural compounds discovered from bacteria during the period from 1940 to 2010.

The period between 1940 and 1980 was recognized as the golden age of drug research, as several drugs were discovered in this era, like antibacterial, antifungal, antiviral, anticancer, antidiabetic, hypercholesterolemia, immunosuppressants, etc. [1,2,3]. In addition to their clinical utility, natural products are invaluable sources for nutrition [4], pigments as natural colors [5], and cosmetics [6]. The advancement of basic biological research to understand health and various diseases [7,8,9] led to the discovery of druggable natural compounds (NCs) from microbes. During the drug discovery era, around 200–300 NCs/year were found, and in the late 1970s, the rate was enhanced to ≈500 NCs/year. After the 1980s, the rate of drug discovery slowed down as it became harder to identify novel and useful NCs from the microbes (Figure 1).

The high rate of re-discovery of NCs discouraged the natural product community [10,11]. Increased use of combinatorial chemistry approaches to develop an analogue of NCs and high-throughput screening of the synthetic small compound libraries for their bioactivity was also a significant reason [12,13]. The other major non-scientific reason for the sluggish pace of drug discovery was the lack of interest among large drug-producing companies [14]. This is due to higher regulatory hurdles related to long-term pre-clinical and clinical studies prior to sale. Another reason is the short time of sale of these drugs until the expiry of their patent and even the brief duration of usage by customers until the expiry date of the drugs. The approximate expenses of producing a new drug are around $1 billion and require 14–15 years [15,16,17]. Even though the growth of drug discovery from natural sources was greatly affected by technical and non-scientific factors, such factors are not in the context of this review article.

### 1.1. Why Novel Drugs Are Required?

Despite several limitations, natural product research should be continued due to unmet needs. Since the 1980s, several new diseases have emerged (e.g., AIDS, Hanta, SARS-CoV, H1N1/09 virus, MERS-CoV, Ebola, Zika virus, yellow fever, etc.), and the currently running pandemic COVID-19 poses a global threat to the human race [18,19]. Increased pathogen resistance to available antibiotics and the toxic effects of certain medicines have triggered the need for novel medicines [20,21]. Instead of discovering novel drugs from natural sources, large drug manufacturers have focused on using combinatorial chemistry during 1990–2005 to generate a library of small compounds [22]. Further efforts were made to screen these compounds for their bioactivity in a high-throughput manner [23]. Unfortunately, success was not achieved due to several reasons, such as difficulty in synthesizing chemically diverse, high-quality libraries of molecules with desirable features of natural products, such as diverse functionality, significant skeletal diversity, and good pharmacokinetic properties, from readily available and inexpensive building blocks [24,25]. By adopting the combinatorial chemistry screening approach, researchers could not succeed in creating diverse and pharmacologically active compounds (Table 1) [26]. Therefore, once again, the researchers have realized the importance of small bioactive compounds from natural sources in drug discovery. Although several drugs were discovered during the slow growth phase of drug discovery, the NCs discovered during this phase were not sufficient to meet the demands of global healthcare challenges [27,28,29]. Therefore, the untapped sources of the novel NCs need to be explored through the use of cutting-edge techniques.

### 1.2. Conventional and Current Strategies for Drug Discovery

The conventional way of discovering drugs from microbes is time-consuming, expensive, and laborious. The separation and purification of microbial secondary metabolites is a critical step and needs expertise. Further, accurate identification of the purified compounds is also manual and requires several analytical techniques, like nuclear magnetic resonance spectroscopy (NMR), infrared spectroscopy (IR), liquid chromatography-mass spectrometry (LC-MS), etc., which eventually slows down the pace of drug discovery. Presently, system biology techniques such as genomics and bioinformatics tools have the potential to enable researchers to understand the arrangement and regulation of secondary metabolite-encoding genes and their network in the microbial genome [30]. Further, the fusion of natural product screening with high-throughput analysis, genomics, chemistry, metabolomics, and microbial biodiversity exploration may offer great opportunities for drug discovery [31,32]. High-throughput screening techniques allow the quick identification of hundreds of samples, while genomics and metabolomics approaches help in understanding the detailed information about the genetic and metabolic potential of the microbes.

Further advancements in the upcoming technologies, such as next-generation sequencing (NGS), artificial intelligence (AI), and machine learning (ML), could further revolutionize the field of microbial drug discovery. These technologies could potentially automate and streamline many of the laborious and time-consuming steps in the drug discovery process, which will eventually make the entire process more efficient and cost-effective. Traditional methods may overlook novel drug candidates, but modern techniques could enhance their discovery. Thus, the future of microbial drug discovery looks promising, with the potential to yield a wealth of new therapeutics for a variety of diseases.

### 1.3. Novel Sources: Novel Drug Approach

The large-scale identification of novel bacterial species in the microbial world, followed by biochemical screening to identify NCs, has always been an opportunity for researchers worldwide. But, exploring novel microbial species for specific NCs or detecting novel NCs from microorganisms has always been a challenging mission. As an alternative, combinatorial libraries comprised of synthetic compounds depict a diverse range of scaffolds with minimal efforts for drug screening. But, in the laboratory, high-throughput screening of the combinatorial libraries gives a lower hit rate than natural compound libraries [33,34]. It has been realized that the role of combinatorial chemistry in drug discovery has remained just to assist the strategies involved in natural product discovery, not to replace them [35]. Considering past research experiences, it has been clearly evidenced that novel sources were always explored for new drugs, but this approach could not sustain the pace of current drug discovery strategies and the global need for drugs. Therefore, the past consequences in drug discovery trigger the researchers to rethink exploring the available microbial sources with hidden cryptic genes for novel drugs. 

Earlier, drug discovery was based on the screening of NCs from source microorganisms, e.g., *Streptomyces* sp., whereas after the discovery of biosynthetic genes in the late 1970s, researchers came to know the genetic basis of the synthesis of NCs [36]. A microbial strain generally produces many NCs; e.g., strains of *Micromonospora* sp., *Streptomyces* sp., *Myxococcus xanthus*, *Aspergillus ochraceus*, and *Sphaeropsideles* sp. were found to produce 50, 12, 38, 16, and 19 compounds, respectively [37,38,39]. The bacterial genes responsible for producing NCs are present in the form of clusters known as biosynthetic gene clusters (BGCs). Profiling of genomic sequences facilitated the expansion of several gene clusters [40,41] and potential drug targets [42,43], which gave hope that genomics could resolve the pharmaceutical productivity crisis. Several bacterial species have more BGCs, like *Streptomyces* sp., while *Ktedonobacteria* sp. consists of 30 and 104 BGCs, respectively [44]. In another study, induced expression of BGCs in Aspergillus nidulans using the transcriptional regulator LaeA resulted in the expression of several novel NCs (e.g., 14 non-ribosomal peptides, two indole alkaloids, 27 polyketides, and one terpene), which were absent in the control species of *A. nidulans*, underscoring the importance of BGCs in the production of diverse NCs [45].

Using the recent bioinformatics tools, more detailed analysis of the BGCs revealed that the bacterial genome contains a trove of silent or cryptic BGCs [46,47,48,49], which are not expressed during normal growth conditions in the laboratory [46]. Indeed, the expression of a large part of microbial BGCs varies with specific environmental or cultural conditions [50]. Recent studies in genome mining have disclosed that microbial genomes comprise a large number of obscure silent BGCs [44,45]. This shows that the bacterial genome contains a trove of silent BGCs, and that could be a gold mine for novel drug discovery. Therefore, in order to identify novel NCs from the silent BGCs, this review paper discusses a strategic workflow comprised of the latest strategies and techniques that enable the high-throughput fashion screening of novel NCs from bacterial species.

## 2. Microbial Genome Mining for Cryptic BGCs

Genome mining is an appropriate method to evaluate the secondary metabolite potential of microorganisms. Genome mining is carried out with the help of bioinformatics tools for the identification and characterization of gene clusters associated with the biosynthesis of NCs. The availability of a large number of bacterial genome sequences publicly is a great alternative to finding the large number of BGCs encoded for the diverse and novel NCs [51]. The strains of unculturable bacterial species are the most untainted source for the genome mining and identification of novel NCs [52]. 

Within the BGCs, a significant portion of the microbial genome comprised genes of different enzymes such as polyketide synthase (PKS) and non-ribosomal peptide synthetase (NRPS), or mixed PKS-NRPS. Gene clusters of PKS/NRPS are particularly found within bacterial species of Proteobacteria, Actinobacteria, Firmicutes, and Cyanobacteria. However, actinomycetes stand out as prime examples of a ‘productive genome’, given their potential to produce a variety of bioactive compounds [53,54]. Polyketides (PKs) and non-ribosomal peptides (NRPs) are a diverse group of bioactive secondary metabolites that are synthesized by PKS and NRPS, respectively. To date, more than 23,000 NCs derived from PKs and NRPs have been identified [55] with a huge spectrum of biological activities such as anticancer (doxorubicin and mithramycin) [56], antibiotics (tetracyclines, erythromycin, and rapamycin, daptomycin), [57,58] immunosuppressants (FK506) [59], and the NRP/PK hybrid compound epothilone is an antitumor agent [60].

Nowadays, several bioinformatics tools are available that are helpful in genome mining for silent BGCs from potent microbes [61]. To perform the bacterial genome mining, we first need the desired genome sequence of the interested microorganism. Bacterial genome sequences can be downloaded from authentic online centers, e.g., the national center for biotechnology information (NCBI) database (http://www.ncbi.nlm.nih.gov) and the Joint Genome Institute (JGI) database (http://jgi.doe.gov), in the form of GenBank, EMBL, or FASTA files. Once the genome sequence is obtained, the gene clusters associated with the biosynthesis of a secondary metabolite can be identified and characterized by using different bioinformatics tools [62,63].

### 2.1. Bioinformatics Tools for Genome Mining

To date, several bioinformatics tools have been developed for searching metabolic gene clusters, such as antiSMASH (Antibiotics and Secondary Metabolite Analysis Shell) [64], SMURF (Secondary Metabolite Unique Regions Finder) (http://www.jcvi.org/smurf) [65], NP.searcher (Natural Product Searcher) (https://dna.sherman.lsi.umich.edu/) [66], CLUSEAN (Cluster Sequence Analyzer) (https://bitbucket.org/tilmweber/clusean/src/master/) [67], ClustScan (Cluster Scanner) [68], MIDDAS-M (Motif Independent De Novo Detection Algorithm for Secondary Metabolite Gene Clusters) [69], CASSIS (Cluster Assignment by Island of Sites) [70], SMIPS (Secondary Metabolites by Inter ProScan) (https://www.ebi.ac.uk/interpro/search/sequence/) [70], software, and C-Hunter (http://fcg.tamu.edu/C_Hunter/) [71]. The numerous novel NCs expressed by BGCs are currently in use as antibiotics (tetracyclines, erythromycin, rapamycin, and daptomycin), immunotherapy (FK506), anticancer (doxorubicin and mithramycin) agents, etc. [72,73,74].

Since 2011, antiSMASH has been helping scientists with their genome mining projects. Currently, antiSMASH is the most trusted and widely used bioinformatics tool, with the additional key features incorporated by CLUSEAN, the NRPS predictor, and Cluster Finder, making it useful for identification and characterization of gene clusters responsible for the biosynthesis of secondary metabolites [75,76]. The current version, antiSMASH 5.0, contains 6200 full annotated and 18,576 drafts of the bacterial genome [77]. The antiSMASH is capable of identifying the BCGs, PKS, NRPS terpenes, siderophores, nucleosides, bacteriocins, beta-lactams, butyrolactones, melanin, antibiotics, and metabolites belonging to other classes [64].

In the current version of antiSMASH 5.0, new detection rules for gene clusters have been added. They improved the prediction of PKS, annotation of resistance genes, gene ontology annotation, and ‘new region’ concept. The antiSMASH 5.0 version encompasses new features for the detection of gene clusters encoded for different secondary products such as acyl-amino acids, β-lactones, C-nucleosides, polybrominated diphenyl ethers, and lipolanthins [78]. Villebro et al. examined the microbial genome using the antiSMASH 5.0 advance module for the identification of biosynthetic gene clusters, specifically for the PKS gene [79]. The study involved the detection and classification of particular genes or proteins in PKS that are responsible for polyketide synthesis. The module is capable of predicting the presence of aromatic polyketide, including possible starter units. A number of elongated malonyl moieties during PKS synthesis will also give clues about the class and molecular weight of the product. Moreover, the module will predict the cyclization patterns present in the product with high accuracy [79]. By using antiSMASH, BGCs encoded for the synthesis of new antibiotics such as pseudopyronine A and B were investigated in the genome of *Pseudomonas putida* BW11M1 [80]. Thus, the cluster-finder algorithm of antiSMASH 5.0 helps in BGC prediction and is capable of finding the signature biosynthetic genes encoded for specific enzymes involved in secondary metabolite biosynthesis. In addition, antiSMASH provides knowledge about PKS/NRPS domain analysis and annotation, substrate specificity, prediction of the core chemical structure of PKS/NRPS, secondary metabolism, protein family analysis, and gene cluster comparative analysis [81].

#### Workflow of antiSMASH

antiSMASH can be operated by using a web server (https://antismash-db.secondarymetabolites.org/) or it can be run as an independent version on a quality-grade desktop computer. 

**Figure 2:**
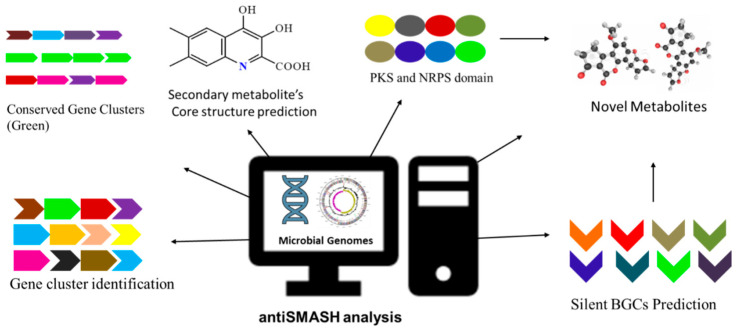
Flowchart of bacterial genome mining using antiSMASH for detection of biosynthesis gene clusters.

For the identification of microbial silent BGCs or gene clusters encoding the biosynthesis of secondary metabolites, a standardized workflow of antiSMASH is shown in Figure 2. For the genome sequence analysis, antiSMASH accepts multiple data formats, such as GenBank, FASTA, or EMBL, as an input file. This tool also retrieves the data from NCBI when the accession number is known. The precision of the antiSMASH data analysis is strongly dependent on the genome sequence. This tool cannot predict the gene clusters in the sequences that involve a large number of small contigs. After the submission of the job at the homepage (https://antismash.secondarymetabolites.org), gene cluster prediction starts, and under the normal server condition, the entire analysis is completed in 0.5–2 h. For the typical bacterial genome, gene cluster prediction analysis may take several days. After completion of sequence analysis, results are available to download from the antiSMASH server [82].

## 3. Strategies to Activate the Expression of Silent BGCs

Silent or cryptic BGCs can be identified bioinformatically, but it is difficult to induce their expression under normal growth conditions in the laboratory. The production of secondary metabolites in microorganisms is accomplished by the expression of BGCs in a controlled manner [83,84,85,86]. The silent or cryptic BGCs in bacteria are not expressed in normal growth conditions [46]. Indeed, the expression of a large part of microbial BGCs varies with specific environmental or cultural conditions [50]. The scientific community has recognized the importance of silent BGCs, and hence various strategies have been established to induce the expression of silent gene clusters. 

The major strategies that include the expression of BGCs are ribosome engineering [87], co-culture screening [88], heterologous host [89], reporter-based selection of mutants [90], overexpression of regulatory proteins [91], and insertion of constitutive/inducible promoters [92,93,94]. These methods were effectively applied in order to induce the expression of silent BGCs in many species. The involvement of complex genetic manipulations during ribosome engineering and the insertion of constitutive/inducible promoter methods [95] are the challenges encountered. The typical culture conditions required for a certain type of bacterial group during co-culture screening affect the expression of BGCs, making this technique unsuitable for natural product research [96]. Complex molecular studies are involved in reported guided mutant selection and heterologous expression. Consequently, these techniques are unfit for the throughput screening of NCs (they are not in the context of this article) [97]. Here, we will focus on a new strategy called HiTES (high-throughput elicitor screening), which enables the expression of the cryptic metabolites in a high-throughput fashion [98,99,100,101].

### 3.1. High-Throughput Expression of Silent BGCs

Elicitors are small compounds available in the library format used for activating the expression of silent BGCs. The HiTES technique is an elicitor screening method that identifies specific small chemical compounds from the library necessary for eliciting the cryptic BGCs. The HiTES method mainly includes the following two steps: the activation of the BGCs by elicitor screening, followed by the detection of newly expressed cryptic metabolites using a genetic readout reporter assay. In this approach, a reporter gene such as LacZ [95] or eGFP [102] is inserted within the BGCs, and the subsequent strain is cultured in a 96-well plate. The microbial culture plate is incubated with a small compound library comprised of different compounds (elicitors) in each well.

**Figure 3:**
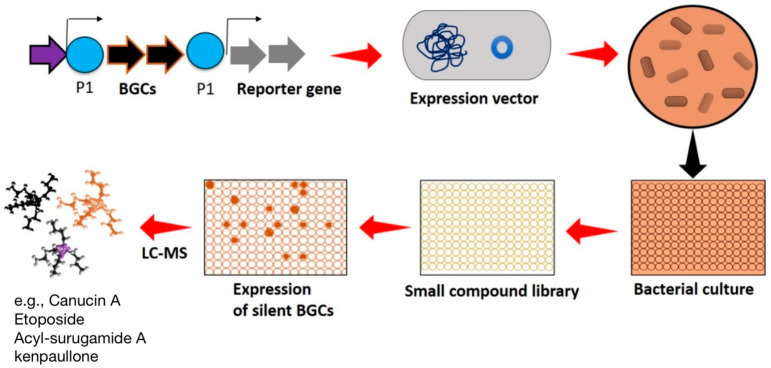
High-throughput elicitor screening (HiTES) technique to activate the silent BGCs. Genetic constructs contain reporter genes used to indicate the expression of cryptic BGCs in the microbes.

After incubation, if the elicitor promotes the growth of the strain, the observed color or fluorescence in the culture plate will highlight the importance of high-throughput screening of small compound elicitors in enhancing the activity of the silent BGCs (Figure 3). HiTES enables the expression of the cryptic gene clusters in different cultural conditions at a time (500–1000). Millions of small compounds in the library are available to screen as an elicitor for inducing the expression of microbial cryptic BGCs. The commercially available small compound libraries are ChemDiv (https://www.chemdiv.com/complete-list/), MolPort (https://www.molport.com/shop/libraries-collections), and Screen-Well^®^ (https://www.enzolifesciences.com/BML-2865/screen-well-natural-product-library/). The HiTES technique enables the researchers to induce the expression of silent BGCs in a rapid manner and also provides insight into the regulation of these gene clusters. 

The major drawback of the HiTES technique is relying on the genetic construct to readout the expression of NCs. The methodology does not include the structural recognition of expressed cryptic metabolites; therefore, users cannot predict novel or already known compounds. Thus, the HiTES technique does not link the bioactivity of the cryptic metabolites; therefore, activity assays need to be conducted upon the discovery of cryptic compounds. The aforementioned drawbacks of the HiTES limit the use of this technique in the high-throughput screening of natural products.

#### 3.1.1. Imaging Mass Spectrometry in High-Throughput Screening of NCs

Recently, Xu et al. amended HiTES as a genetic-free technique by removing the genetic construct used to read the expressed cryptic metabolites in the microbial culture [103]. In the genetic-free HiTES technique, expression of silent BGCs is triggered by deploying the bacterial culture in hundreds of different (500–1000) cultural conditions (small compound elicitors) at a time.

The HiTES technique coupled with laser ablation electrospray ionization-imaging mass spectrometry (LAESI-IMS) is referred to as ‘HiTES-IMS’. Subsequent analysis of the expressed metabolites is carried out in a high-throughput manner using laser ablation electrospray ionization coupled with imaging mass spectrometry (LAESI-IMS). The latest advances in the IMS technique have provided a flexible way for rapid analysis of biological samples for both known and unknown compounds [104]. The IMS analysis requires a very small quantity of samples and provides the facility to analyze specific compounds. There are currently several IMS techniques available to identify NCs [104], but the LAESI coupled with mass spectroscopy (MS/MS) is a relevant technique to detect NCs directly from biological samples in a high-throughput fashion [50]. 

**Figure 4:**
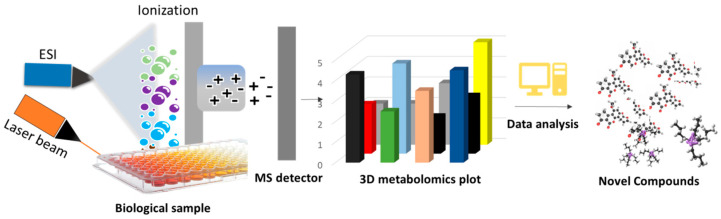
HiTES coupled with laser ablation electrospray ionization-imaging mass spectroscopy (LAESI-IMS) technique for the high-throughput identification of the novel NCs directly from the microbial culture plate.

The LAESI-IMS is a newly emerged technique in which the biological sample absorbs a mid-infrared laser (ÿ = 2.94 μm), creating an ablation plume of compounds that are ionized by electrospray and inserted into the mass spectrometer (Figure 4) [105,106,107,108]. This technique is capable of identifying a broad range of molecules, including lipids, peptides, alkaloids, phenolics, and several other types of compounds [109]. The LAESI-IMS method has been validated effectively on several biological samples belonging to bacteria, plants, and fungi [110,111]. This technique uses an ambient ionization method; therefore, samples can be analyzed directly without any prior preparation at atmospheric pressure. Unlike other IMS methods, LAESI often has certain disadvantages, as it is not ideal for analyzing dried samples and cannot differentiate the isobaric ions. However, these problems can be resolved by dissolving the sample in water before imaging [110] and analyzing it using LAESI coupled with MS/MS [111]. 

#### 3.1.2. HiTES Coupled with the IMS Technique in High-Throughput Screening of NCs

The HiTES-IMS technique is an endogenous monoculture strategy for eliciting silent BGCs and detecting the cryptic NCs of a given bacterial strain in a rapid and untargeted fashion. Using HiTES-IMS, screening of novel NCs from known or diverse bacteria has become automated and easier. The HiTES-IMS technique has been effectively validated by inducing the expression of silent BGCs and subsequent identification of cryptic NCs in different bacterial strains belonging to Gram-positive, Gram-negative, and distinct actinomycetes [97]. Regardless of the bacterial species and the genome sequence details, HiTES-IMS can express and identify several cryptic metabolites under different cultural conditions at a time. Compared with conventional genetic-based methods that typically take a couple of weeks to months to express and identify novel cryptic metabolites from a microbial source, HiTES-IMS can do the same job with ultra-accuracy in a few hours or a couple of days. Furthermore, this technique, along with enabling the expression of silent BGCs, may also be effective in enhancing the production of already-recognized bioactive compounds and recognizing the respective elicitor compounds in the library. Li et al. used the LAESI technique for creating 3D images of metabolites by ‘depth profiling’ a bacterial sample [107]. Recently, LAESI-IMS was used to identify novel NCs by screening a library of small compounds (1000 elicitors) to activate the silent BGCs in a throughput manner. They found Canucin A as a novel compound, utilizing kenpaullone as an elicitor [97]. Furthermore, Tomm et al. (2019) described several novel compounds that were identified through the HiTES-IMS technique [98].

##### Workflow of HiTES-IMS

The selected bacterial cultures in 96-well plates were subjected to HiTES with a 502-member natural-product library [112]. After proper incubation time, the expression of NCs is analyzed using LAESI-IMS directly from the sample plate. The LAESI-IMS generates a raw data file of induced or enhanced metabolites, and the file format varies with the individual manufacturer’s software used. Furthermore, each company’s software generates its own raw data file types that do not lend themselves to open-source software platforms. To address this problem, an open and standard format for IMS datasets has been implemented in the imzML format [113]. The data analysis software of LAESI-IMS extracts the signals of each well above a set cut-off value and depicts the data in both 2D and 3D plots. The *x*-axis of the plot shows the m/z value, and the *y*-axis depicts the intensity of individual NCs expressed in the presence of respective elicitors. This approach allows for the detection of individual compounds, whether their expression is enhanced or triggered by basic visualization. In the presence or absence of elicitors, the metabolomics data produced in individual wells of the 96-well plate can be compared to recognize the induced novel compounds. The same approach can be used to identify the enhanced yield of specific secondary metabolites. Furthermore, ‘dereplication’ is also an accurate and rapid method for distinguishing the known and unknown (novel) compounds within the sample results [114]. The data on the detected metabolites are extracted by MatLab 3D ver. R2024a software and can be exported to an Excel file. The hit compounds can be eventually validated using flask culture followed by HPLC-MS/MS technique.

During the high-throughput screening of natural products, a large amount of mass spectral data is produced by mass spectrometry. This situation becomes challenging, particularly in the high-throughput screening experiments using the HiTES-IMS technique, which directly penetrate hundreds of biological samples at a time and produce an enormous amount of MS/MS raw data. Such data contain both known and unknown MS/MS spectra. Therefore, an accurate and quick analysis of this data is a daunting task. However, the new dereplication techniques currently available have rendered it possible to rapidly identify known MS/MS spectra and molecular networking, accompanied by the annotation of unknown MS/MS data [115,116].

## 4. Dereplication of Natural Products

The process in which structural information about a chemical compound is used for its identification among the sample data is called ‘dereplication’. The dereplication process has been proven to be an essential step in natural product research. The dereplication method enables the rapid detection of chemical compounds from the crude MS data during the screening of natural products [117]. Thus, dereplication enhances the performance of high-throughput screening of natural products [118]. Several databases, such as PubChem (≈83 million compounds), ChemSpider (≈58 million), ChEMBL (≈2.1 million), ChemBank (≈1.2 million), ChEBI (440,000), SciFinder (≈161 million), and several other libraries, are available with the structural information of millions of chemical compounds [119]. This approach eventually eliminates the re-isolation and structural identification processes [117].

In recent years, the development of several useful bioinformatics tools has been assisted by the process of dereplication through the identification of known compounds from the huge crude MS/MS data [120,121]. These tools identify the NCs by matching the MS data generated from the experiment to the MS data repositories. 

To assist the rapid screening of NCs, dereplication procedures usually consider the combination of various separation strategies, spectroscopy, and database searching methods [119]. The dereplication of NCs can be achieved best with the help of a computer-based strategy called molecular networking (MN). The MN-based dereplication comprised the visualization and interpretation of complex MS/MS data for the rapid identification of the known compounds and molecular networking of the unknown mass spectra in crude MS/MS data [122]. The MN consists of two crucial steps, i.e., (1) organization and visualization of MS/MS datasets and (2) automation in database search. The key strategies for MN-based dereplication include the following: (1) integration of MS data; (2) harnessing mass shift differences; and (3) integration of functional annotation. The integration and visualization of tandem MS data are being achieved based on the similarity of the spectral map. For structurally similar compounds, nodes of similar fragmentation spectra would cluster together and generate the cluster of analogues [123]. The MN utilizes the harnessing of mass shift differences and exploits mass-to-charge (m/z) ratios between related molecules [122]. This strategy facilitates the profiling of meta-mass shift chemicals that allow the identification of known chemical groups and reveal the specific biochemical transformation [124]. 

More recently, researchers have used the algorithm tool DEREPLICATOR+ (http://mohimanilab.cbd.cmu.edu/software/), which utilizes spectral networks for the high-throughput identification of variants of known natural products. Among the several available tools, to date, the DEREPLICATOR+ tool has gained wide acceptance in the high-throughput screening of natural products [117]. It has been reportedly improved and facilitates the identification of polyketides, terpenes, benzenoids, alkaloids, flavonoids, etc.

### Workflow to Generate MN

**Figure 5:**
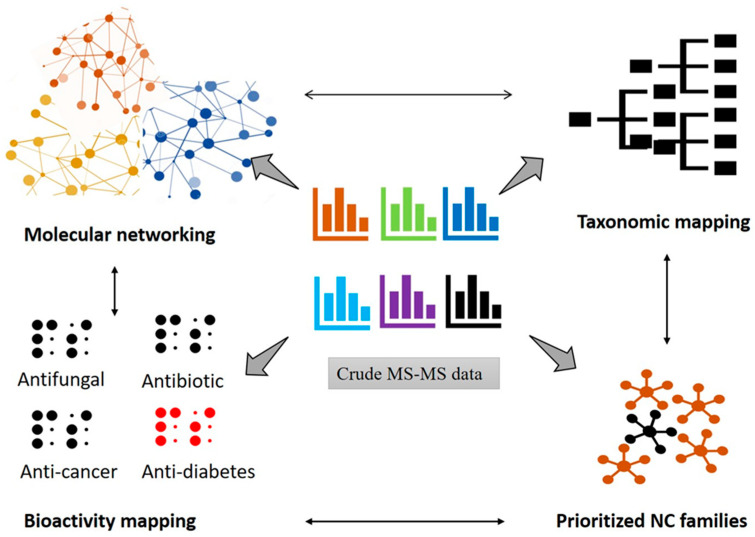
Various applications of crude MS-MS data in GNPS for the generation of molecular networking (MN).

The workflow that involves MN-based dereplication is schematically represented in Figure 5. MN is a computer-based approach that extracts and arranges the experimental MS/MS data according to their spectral similarities [125]. Global Natural Product Social (GNPS) is currently the most widely used and accepted online platform for MN (https://gnps.ucsd.edu/ProteoSAFe/static/gnps-splash.jsp). The basic process for producing MN utilizing raw data involves data collection, data file conversion, GNPS-MassIVE upload, MN generation commands, and network analysis accompanied by visualization. For developing a molecular network, the first step is the collection of MS/MS spectra. The file format of the raw data needs to be converted to an open format such as mzXML, mzML, or MGF. These open file formats are accessible on the GNPS-MassIVE platform. After submission of the MS/MS datasets, the GNPS program identifies the known compounds and treats the unknown compounds to generate a molecular network using the spectral similarity of the datasets. The generation of a molecular network can be achieved based on the cosine scores of MS/MS spectra [126]. Basically, cosine scores measure the relatedness in MS/MS spectra. Furthermore, the MS/MS spectral data needs to be converted to mzXML format, which is a text-based format of MS/MS data [123,127]. Finally, MN can be analyzed and visualized using GNPS, but Cytoscape, an open-source platform, can also be used [124]. The generated text file can be imported into Cytoscape to visualize the molecular network. Using Cytoscape, identical molecules in the analogue or compound family can be explored visually.

## 5. Conclusions

In concurrent efforts to bring the antibiotic era back, success in natural product research does not end with the identification of novel NCs from natural sources. The researchers need to modify their routine approaches to screen NCs in a high-throughput fashion. Recently, microbial silent BGCs have emerged as an opportunistic trove for the mining of novel NCs. The interdisciplinary techniques discussed here, such as antiSMASH, HiTES, and LAESI-IMS, are the latest and validated for their usage in natural product research. The strategic workflow described here begins with the identification of the potential microbial strain containing a large reservoir of silent BGCs, activation followed by the expression of silent BGCs, and finally the identification of novel NCs in a high-throughput fashion. Further, dereplication strategies enhance the rapid identification of known compounds and the generation of a molecular network of unknown MS/MS spectra on the GNPS platform. The sequential application of these strategies will eventually enable the rapid screening of novel NCs.

## Figures and Tables

**Table 1:** Comparative analysis of natural compound discovery from various sources [26].

	Number	%	Drugs	Success%
Synthetic compounds	8–10 M	93–94%	2000–2500	0.005%
All natural compounds (plants + animals + microbes)	≈500,000	4.7–5.8%	1200–1300	0.6%
Microbial compounds	≈70,000	0.66–0.82%	450–500	1.6%

## Data Availability

Data will be provided upon individual request.
